# Identification of T‐cell exhaustion‐related gene signature for predicting prognosis in glioblastoma multiforme

**DOI:** 10.1111/jcmm.17927

**Published:** 2023-08-27

**Authors:** Yue‐hui Liu, Hong‐quan Jin, Hai‐ping Liu

**Affiliations:** ^1^ Department of Neurology Affiliated Hospital of Inner Mongolia Minzu University Tongliao China; ^2^ College of Life Science and Food Engineering Inner Mongolia Minzu University Tongliao China

**Keywords:** glioblastoma multiforme, molecular classification, risk stratification, subtypes, T‐cell immunity

## Abstract

Glioblastoma multiforme (GBM) is a highly malignant primary brain tumour with a poor prognosis in adults. Identifying biomarkers that can aid in the molecular classification and risk stratification of GBM is critical. Here, we conducted a transcriptional profiling analysis of T‐cell immunity in the tumour microenvironment of GBM patients and identified two novel T cell exhaustion (TEX)‐related GBM subtypes (termed TEX‐C1 and TEX‐C2) using the consensus clustering. Our multi‐omics analysis revealed distinct immunological, molecular and clinical characteristics for these two subtypes. Specifically, the TEX‐C1 subtype had higher infiltration levels of immune cells and expressed higher levels of immune checkpoint molecules than the TEX‐C2 subtype. Functional analysis revealed that upregulated genes in the TEX‐C1 subtype were significantly enriched in immune response and signal transduction pathways, and upregulated genes in the TEX‐C2 subtype were predominantly associated with cell fate and nervous system development pathways. Notably, patients with activated T‐cell activity status in the TEX‐C1 subgroup demonstrated a significantly worse prognosis than those with severe T cell exhaustion status in the TEX‐C2 subgroup. Finally, we proposed a machine‐learning‐derived novel gene signature comprising 12 TEX‐related genes (12TexSig) to indicate tumour subtyping. In the TCGA cohort, the 12TexSig demonstrated the ability to accurately predict the prognosis of GBM patients, and this prognostic value was further confirmed in two independent external cohorts. Taken together, our results suggest that the TEX‐derived subtyping and gene signature has the potential to serve as a clinically helpful biomarker for guiding the management of GBM patients, pending further prospective validation.

## 1 INTRODUCTION

Brain and other nervous system tumours (CNS) are a major cause of cancer‐related deaths in males younger than 40 and females younger than 20 years, with 25,050 new cases and 18,280 deaths reported in the United States, according to the latest cancer statistics report.^1^ Among CNS tumours, glioblastoma multiforme (GBM) is the most common type in adults, accounting for approximately 48.6% of all malignant central nervous system tumours.^2^ While surgical resection followed by radiotherapy and chemotherapy is the standard treatment for GBM, it remains a highly aggressive and lethal disease. Patients frequently develop drug resistance leading to local recurrence and have an abysmal prognosis with a 5‐year survival rate of just 5%.^3^, ^4^, ^5^, ^6^ Therefore, identifying biomarkers for early detection and prognostication is a pressing clinical imperative.

The rapid development of high‐throughput sequencing in the past two decades has enabled an enhanced understanding of the molecular mechanisms involved in GBM tumour initiation, progression, recurrence and therapeutic resistance.^7^, ^8^, ^9^ The high molecular heterogeneity of GBM has been extensively characterized at the genomic, transcriptomic and epigenetic levels, resulting in the refinement of histopathological classification and identification of novel subtypes based on transcription, genetic alteration, and DNA methylation.^9^, ^10^, ^11^, ^12^, ^13^ A recent single‐cell omics study has provided unprecedented insights into the intratumoral heterogeneity of tumours, which has important clinical implications for guiding diagnosis, prognosis, and therapeutic recommendations.^14^, ^15^, ^16^, ^17^ T cell exhaustion (TEX) is a state of T cell dysfunction characterized by poor effector function, sustained inhibitory receptor expression, and altered transcriptional state distinct from that of functional effector or memory T cells.^18^ Advancements in the understanding of tumour‐immune microenvironment have highlighted the significance of T cell exhaustion (TEX) in the prognosis and therapeutic efficacy of different tumour types.^19^ Despite the extensive characterization of TEX in recent studies,^20^, ^21^, ^22^ a simple and robust gene signature derived from TEX for predicting GBM prognosis is yet to be explored in detail.

In this study, we aimed to investigate the association between TEX and survival outcome in GBM by analysing TEX profiles. Additionally, we aimed to develop a TEX‐based subtyping and gene signature to improve molecular classification and risk stratification of GBM patients.

## 2 MATERIALS AND METHODS

### 2.1 GBM patient datasets

This study included three GBM cohorts, including 167 samples with RNA‐seq data and clinical information from The Cancer Genome Atlas (TCGA) project (https://xena.ucsc.edu/), 80 samples with microarray data profiling by Affymetrix Human Genome U133 Plus 2.0 Array and clinical information from Gene Expression Omnibus under accession number GSE7696 (www.ncbi.nlm.nih.gov/geo/query/acc.cgi?acc=GSE7696),^23^ and 693 samples from Chinese Glioma Genome Atlas (CGGA) database (http://www.cgga.org.cn/).^24^ We conducted a discovery and validation study in which the TCGA cohort was used as a discovery cohort, and the GEO and CGGA cohorts were used as external independent cohorts for validation.

### 2.2 Construction of a TEX‐related signature

The univariate and multivariate Cox proportional hazards regression analysis was used to evaluate the association between genes and overall survival for identifying independent prognostic genes. The feature selection procedure with the stepwise selection method was used using the R package ‘stats’ (v4.2.2) for these prognostic genes to identify optimal gene combinations to construct a predictive model.^25^ Finally, a TEX‐derived gene signature risk scoring model for survival prediction was constructed according to the expression of prognostic genes and using the multivariate Cox regression coefficient as the weight previously described.^26^ The optimal risk cut‐off value of the 12TexSigwere determined using the R package ‘survminer’.

### 2.3 Bioinformatics analysis

Consensus clustering was performed to identify potential subtypes using the R package ‘ConsensusClusterPlus’ (v1.62.0).^27^ Differential expression analysis for mRNA, lncRNA and miRNA between two subgroups was conducted using the R package ‘limma’ (v3.54.1).^28^ Those genes with |log(fold change) > 2| and false discovery rate (FDR) < 0.05 were considered significantly differentially expressed genes. Functional gene sets were derived from the MsigDB database (https://www.gsea‐msigdb.org/gsea/msigdb). Pathway activity score was calculated using the single sample gene set enrichment analysis (ssGSEA) with GSVA (v1.46.0). Tumour‐infiltrating immune cells and tumour immune microenvironment were computationally estimated using the CIBERSORT,^29^ xCell,^30^ EPIC^31^ and MCP‐counter.^32^

### 2.4 Functional analysis

The experimentally validated microRNA‐target relationships were obtained from the miRTarBase database (https://mirtarbase.cuhk.edu.cn/~miRTarBase/miRTarBase_2022/php/index.php). Functional enrichment analysis for targets of mRNAs was used to annotate miRNA function. Pearson correlation coefficient between mRNAs and lncRNAs was calculated to identify co‐expressed mRNA of lncRNAs. Functional enrichment analysis for co‐expressed mRNA of lncRNAs was used to annotate lncRNA function. Functional enrichment analysis was conducted using the R package ‘clusterProfiler’.^33^

### 2.5 Statistical analysis

All statistical analyses were conducted using the R software (V.3.4.4). Mann–Whitney *U* test was used to compare the two groups. Spearman's correlation was used to measure the association between two continuous variables. Kaplan–Meier method and log‐rank tests were used to evaluate the differences in overall survival between different patient groups with R package ‘survival’ (v3.5‐0). Univariate and multivariate Cox proportional hazards regression analysis was performed on the individual variables, and hazard ratios (HR) and 95% confidence intervals (CI) were calculated. A prognostic nomogram was constructed using prognostic genes to predict individual survival outcomes with R package “rms” (v6.4‐1). A value of *p* < 0.05 is statistically significant.

## 3 RESULTS

### 3.1 Transcriptional profiling of TEX‐related genes reveals heterogeneous subgroups of GBM patients

**FIGURE 1:**
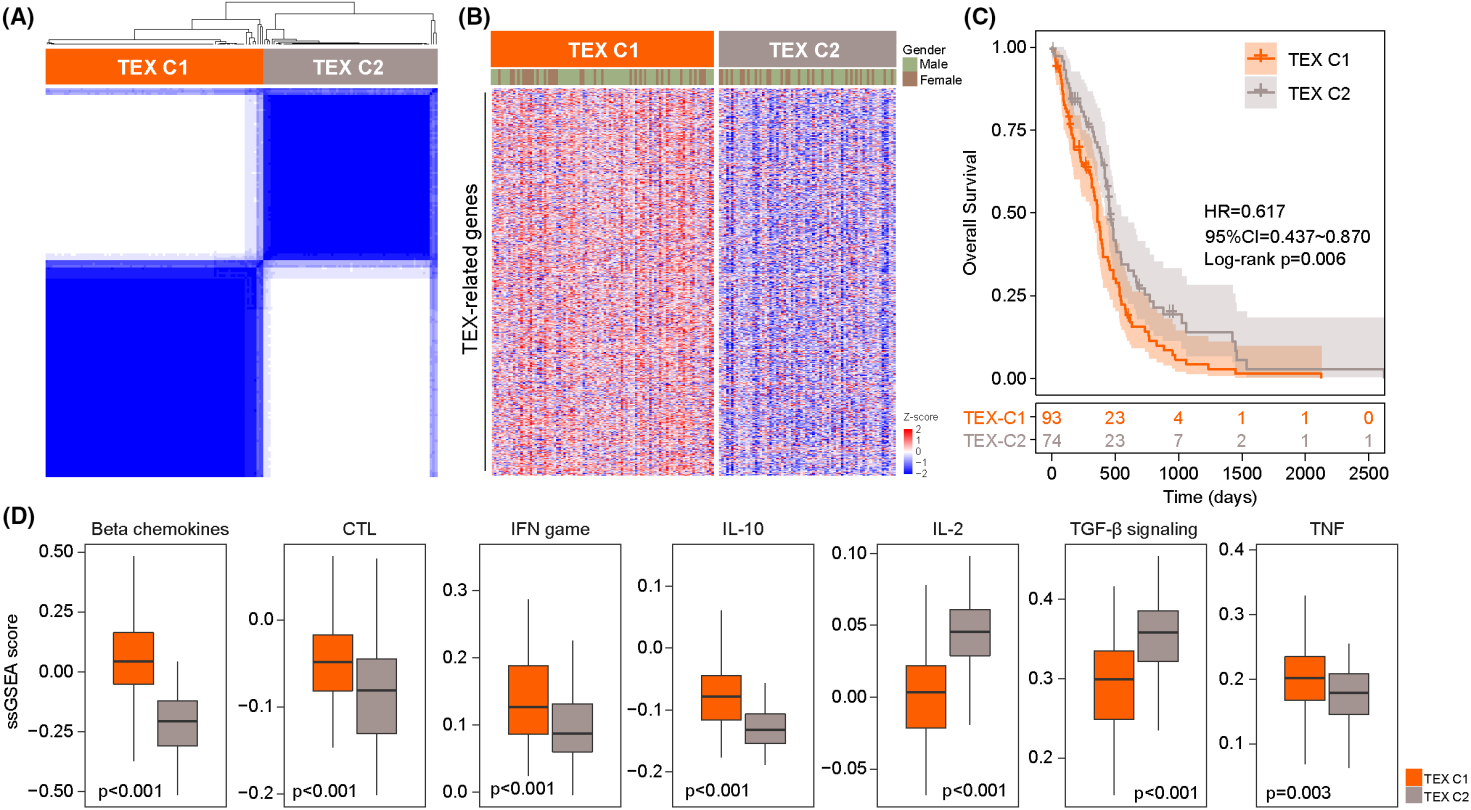
Identifying heterogeneous subgroups of GBM patients based on the expression pattern of TEX genes. (A) Consensus matrix heatmap identifying two clusters (*k* = 2) and their corresponding regions. (B) Expression pattern of TEX genes in the two distinctive subtypes. (C) Kaplan–Meier survival curves of overall survival between two distinctive TEX subtypes. (D) Boxplots showing enrichment analysis of seven T‐cell‐related pathways between two distinctive TEX subtypes.

We collected 563 TEX‐related genes from a previous study.^19^ To explore the TEX heterogeneity in GBM, we performed consensus clustering based on expression levels of 563 TEX‐related genes and identified two clusters, referred to here as TEX‐C1 and TEX‐C2 (Figure 1A,B). As shown in Figure 1C, survival analysis indicated that patients in the TEX‐C2 cluster had a better prognosis than those in the TEX‐C1 cluster (HR = 0.617, 95% CI 0.437–0.870; log‐rank *p* = 0.006) (Figure 1C). In addition, the 1‐ and 3‐year survival rates of the TEX‐C2 cluster were 69.8% and 13.9%, respectively, whereas the corresponding rates in the TEX‐C1 cluster were 47.2% and 4.2%. To test the association between TEX clusters and T‐cell activity status, we conducted ssGSEA analysis to compare the activity status of seven T‐cell related pathways between TEX‐C1 and TEX‐C2 clusters. We found that there was a significantly different T cell activity status between TEX‐C1 and TEX‐C2 clusters. As shown in Figure 1D, there were significantly higher enrichment scores for all seven T‐cell‐related pathways in the TEX‐C1 cluster compared to the TEX‐C2 cluster. These findings suggested that T cell activity status correlated with survival outcomes and that the high‐risk group possessed an activated T‐cell activity status and the low‐risk group possessed severe T cell exhaustion.

### 3.2 Characterization of tumour immune microenvironment of TEX‐based subtypes

**FIGURE 2:**
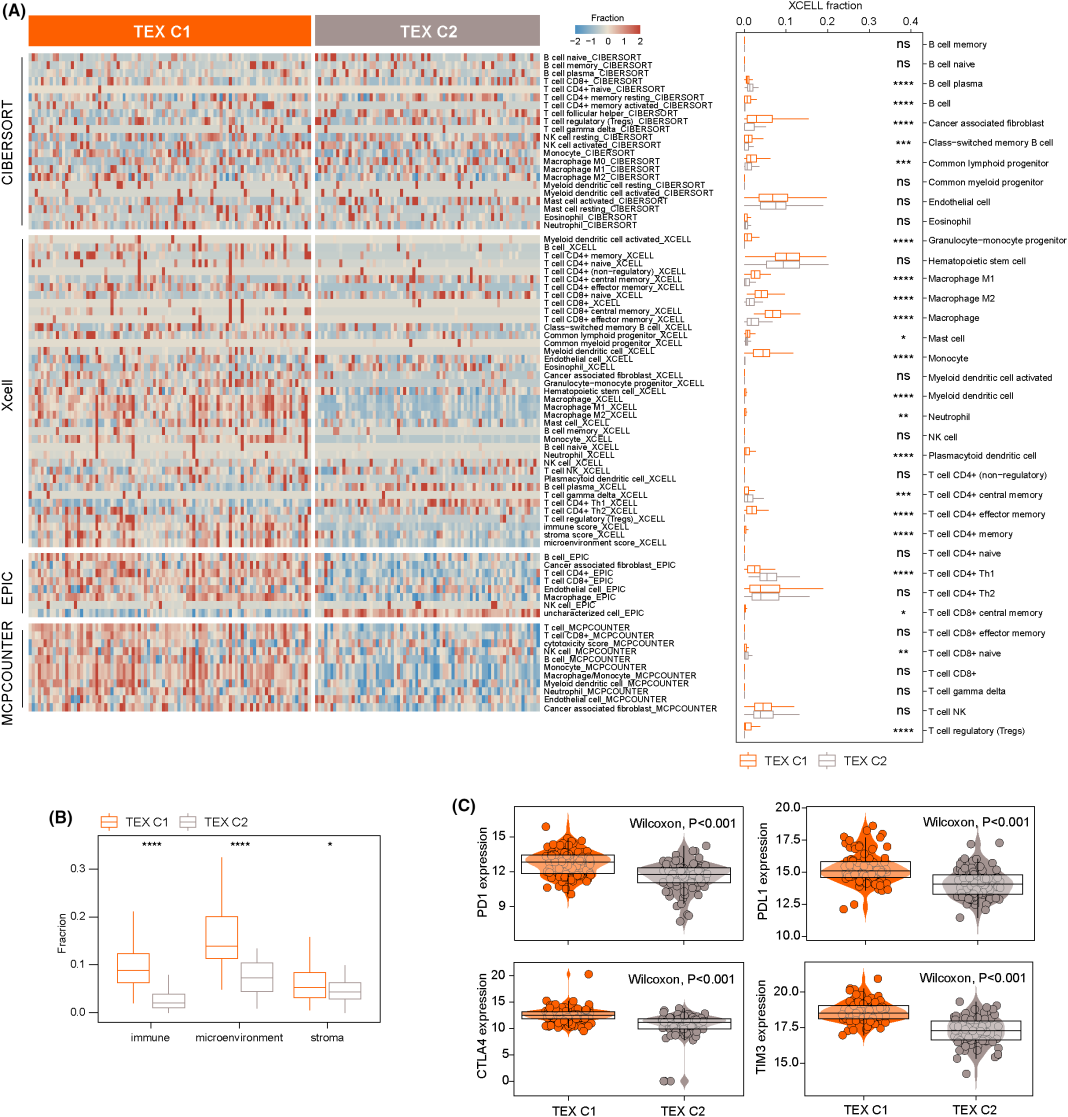
Immune microenvironment characterization of TEX‐based subgroups. (A) The abundance of infiltrating immune cells in the tumour immune microenvironment using CIBERSORT, xCell, EPIC and MCP‐counter. (B) Boxplot showing the differences in the immune score, immune microenvironment score and stromal score between TEX‐based subgroups. (C) Boxplot showing the expression differences of four immune checkpoints between TEX‐based subgroups.

To further characterize the heterogeneity of the tumour immune microenvironment of TEX‐based subtypes, we first estimated the abundance of infiltrating immune cells in the tumour immune microenvironment using CIBERSORT, xCell, EPIC and MCP‐counter. The infiltration levels of immune cells between the two TEX‐based subtypes are shown in Figure 2A. The estimated infiltration levels of immune cells were higher in the TEX‐C1 subtype than in the TEX‐C2 subtype. Further analysis also indicated that the immune score, immune microenvironment score and stromal score are all significantly higher in the TEX‐C1 subtype compared to the TEX‐C2 subtype (Figure 2B). We also compared expression levels of four immune checkpoints between the two TEX‐based subtypes and found that expression of four immune checkpoints (PD1, PD‐L1, CTLA4 and TIM3) is significantly higher in TEX‐C1 subtype than the TEX‐C2 subtype (Figure 2C).

### 3.3 The multidimensional molecular features of TEX‐based subtypes

**FIGURE 3:**
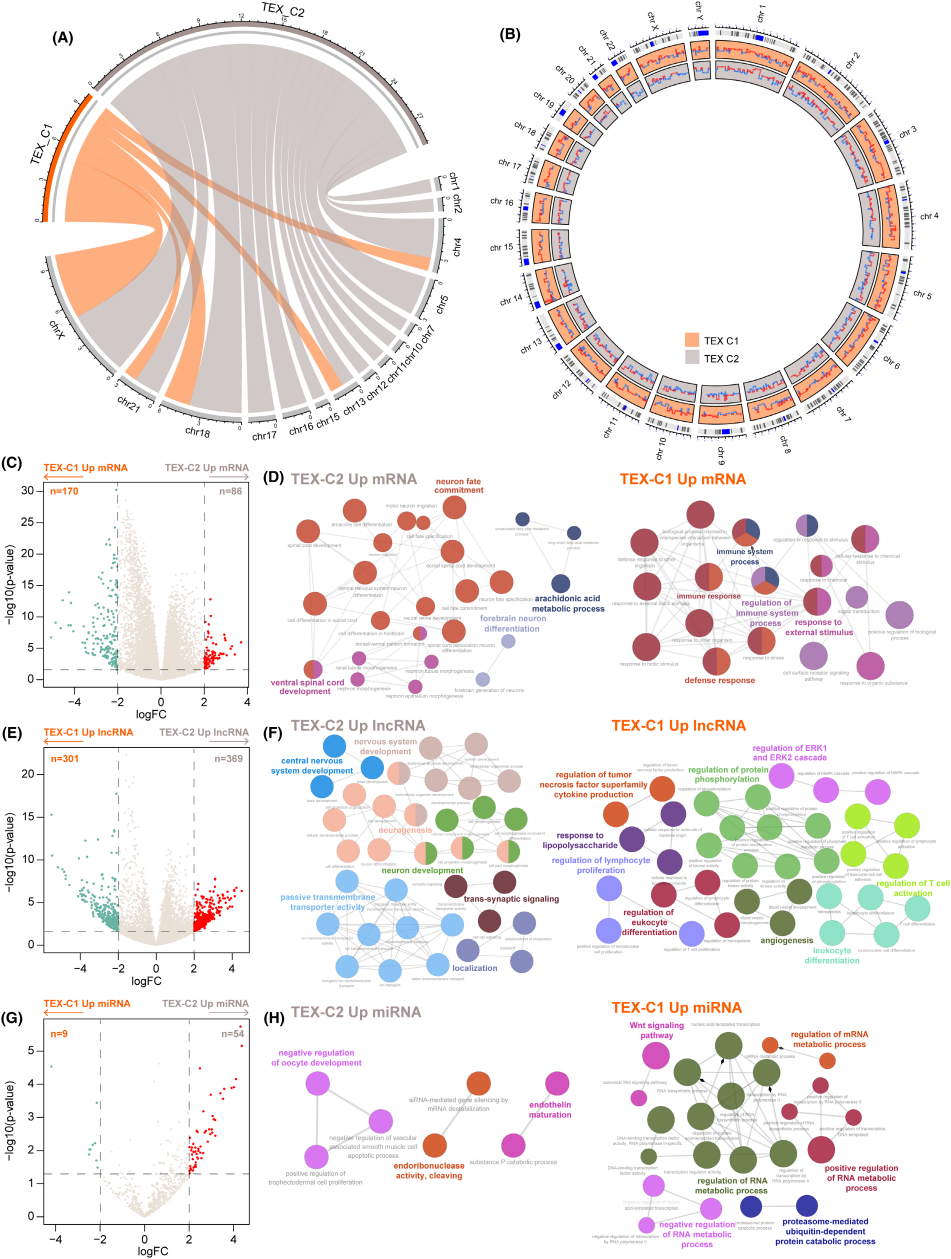
The multidimensional molecular features of TEX‐based subgroups. (A) Circos plot showing the landscape of somatic mutations between TEX‐based subgroups. (B) Circos plot showing the landscape of copy number variation between TEX‐based subgroups. (C) Volcano plot showing differentially expressed mRNAs between two distinctive TEX subtypes. (D) Functional enrichment network of GO terms of differentially expressed mRNAs. (E) Volcano plot showing differentially expressed lncRNAs between two distinctive TEX subtypes. (F) Functional enrichment network of GO terms of differentially expressed lncRNAs. (G) Volcano plot showing differentially expressed miRNAs between two distinctive TEX subtypes. (H) Functional enrichment network of GO terms of differentially expressed miRNAs.

To identify multidimensional molecular features associated with TEX‐based subgroups, we first genomic variation pattern between two TEX‐related subtypes. Mutation analysis showed that mutations on chromosomes 1, 2, 5, 7, 10, 11, 12, 16 and 17 were not observed in the TEX‐C1 subtype, while the mutations of the TEX‐C1 subtype were mainly concentrated on chromosomes 18 and X (Figure 3A). Overall, the TEX‐C2 subtype exhibited a significantly higher number of mutations than the TEX‐C1 subtype. Copy number variation analysis revealed that Copy number variation differed between the two TEX‐related subtypes (Figure 3B).

We next performed a differential expression analysis for three molecular layers (mRNA, miRNA, lncRNA) between TEX‐C1 and TEX‐C2 clusters. We identified 170 mRNAs that are upregulated in the TEX‐C1 cluster, and 86 mRNAs upregulated in the TEX‐C2 cluster (Figure 3C). Functional enrichment analysis demonstrated that genes upregulated in the TEX‐C1 cluster tended to be enriched in immune response and signal transduction, and genes upregulated in the TEX‐C2 cluster tended to be enriched in cell fate, and nervous system development (Figure 3D). For lncRNAs, 301 lncRNAs were upregulated in the TEX‐C1 cluster and 369 lncRNAs upregulated in the TEX‐C2 cluster (Figure 3E). To explore the functional roles of these lncRNAs, we performed a co‐expressed analysis between mRNAs and 370 lncRNAs by calculating the Pearson correlation coefficient. Functional enrichment analysis for mRNAs co‐expressed with lncRNAs found that lncRNAs upregulated in the TEX‐C1 cluster tended to be enriched in immune regulation, including regulation of tumour necrosis factor superfamily cytokine production, regulation of leukocyte differentiation and regulation of T‐cell activation, and lncRNAs upregulated in TEX‐C2 cluster tended to be enriched in nervous system development (Figure 3F). For miRNAs, we identified nine miRNAs overexpressed in the TEX‐C1 cluster and 54 miRNAs overexpressed in the TEX‐C2 cluster (Figure 3G). We obtained their experimentally validated target genes and performed functional enrichment analysis. We found that miRNAs upregulated in TEX‐C2 have an enrichment of regulation of oocyte development and endoribonuclease activity, cleaving, and miRNAs upregulated in TEX‐C1 enriched in regulation of RNA metabolic process and Wnt signalling pathway (Figure 3H).

### 3.4 Identification and development of the TEX‐related signature to predict survival outcome

**FIGURE 4:**
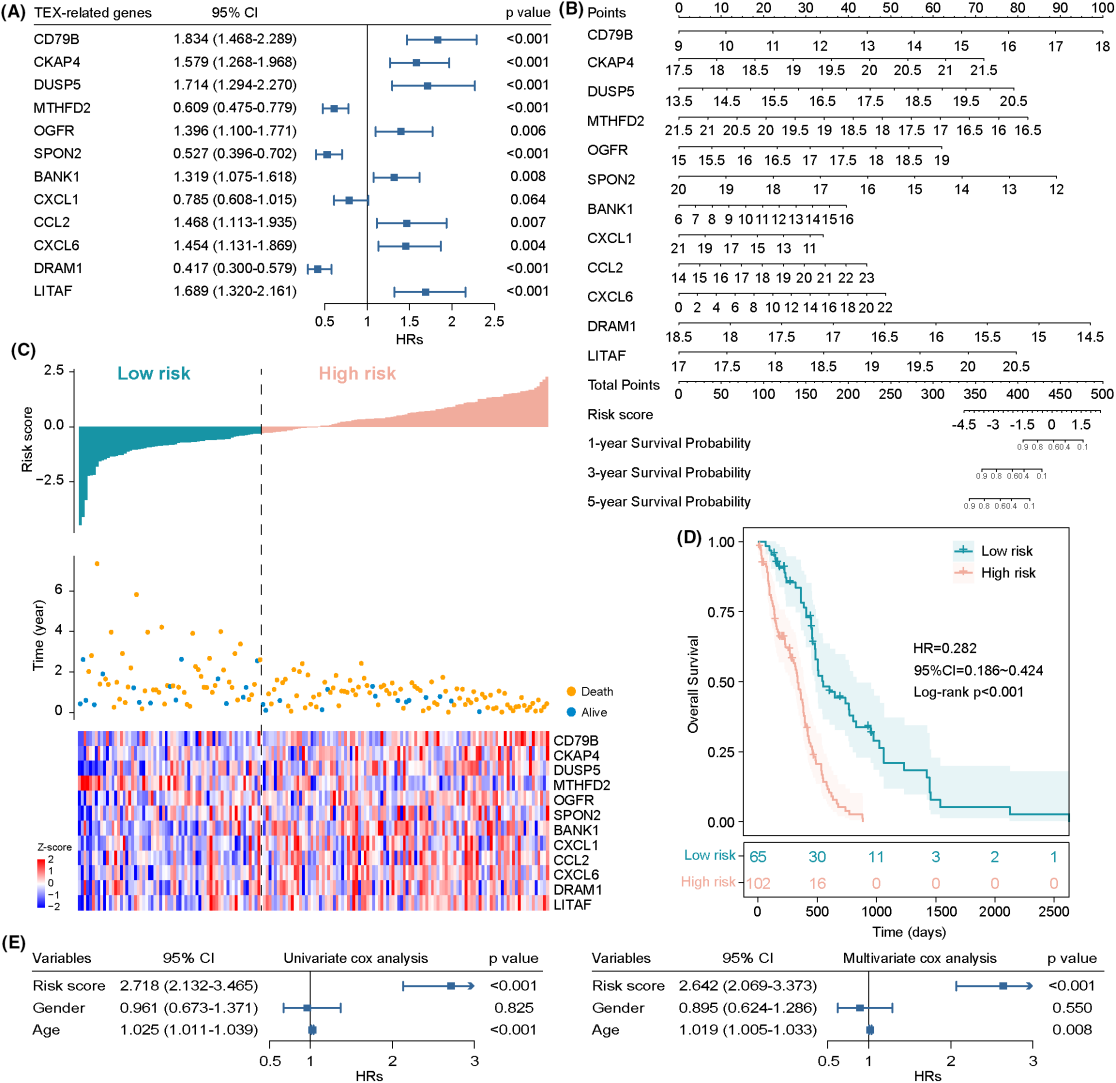
Identification and development of the TEX‐related signature to predict survival outcome. (A) Forest plot showing the results of multivariate analysis for overall survival of 12 TEX genes. (B) A nomogram for predicting 1‐, 3‐ and 5‐year survival rates using these 12 genes. (C) The distribution of 12TexSig risk scores, gene expression profiles and survival status. (D) Kaplan–Meier survival curves of overall survival between two risk groups predicted by 12TexSig. (E) Forest plot showing the results of univariate and multivariate analysis for overall survival of the 12TexSig and other clinical features.

To explore the associations between TEX and the patient's prognosis, we conducted univariate Cox proportional hazards regression analysis for 563 TEX genes with overall survival time. We identified 101 of 563 TEX‐related genes significantly associated with overall survival (*p* < 0.05) (Table S1). To examine the independence of prognostic value, we adopted multivariate Cox proportional hazards regression analysis for 101 prognostic TEX‐related genes with clinical variables (gender and age), and 27 of 101 prognostic TEX‐related genes were found to retain their prognostic significance (*p* < 0.05) (Table S2). To determine the optimal gene combination to construct a predictive model, we performed a feature selection procedure for 27 independent prognostic TEX‐related genes using a stepwise selection method and identified an optimal combination of 12 TEX genes (CD79B, CKAP4, DUSP5, MTHFD2, OGFR, SPON2, BANK1, CXCL1, CCL2, CXCL6, DRAM1 and LITAF) with the most significant predictive ability (Table 1). Of them, four genes (MTHFD2, SPON2, CXCL1 and DRAM1) with HR <1 may be protective factors owing to the close association between their high expression and longer overall survival, whereas the remaining six genes (CD79B, CKAP4, DUSP5, OGFR, BANK1, CCL2, CXCL6 and LITAF) tended to be risky prognostic factors and their high expression was associated with shorter overall survival (Figure 4A).

**TABLE 1:** Detailed information of 12 prognostic TEX‐related genes.

Gene name	Ensembl IDs	Location	Hazard ratios	95% Confidence intervals	*p*‐Value
CD79B	ENSG00000007312	Chromosome 17: 63928738–63932336 reverse strand	1.188	1.041–1.355	0.011
CKAP4	ENSG00000136026	Chromosome 12: 106237881–106304279 reverse strand.	1.49	1.116–1.988	0.007
DUSP5	ENSG00000138166	Chromosome 10: 110497907–110511533 forward strand.	1.245	1.053–1.472	0.01
MTHFD2	ENSG00000065911	Chromosome 2: 74186172–74217565 forward strand	0.698	0.561–0.869	0.001
OGFR	ENSG00000060491	Chromosome 20: 62804835–62814000 forward strand	1.443	1.007–2.07	0.046
SPON2	ENSG00000159674	Chromosome 4: 1166932–1208962 reverse strand.	1.175	1.023–1.351	0.023
BANK1	ENSG00000153064	Chromosome 4: 101411286–102074812 forward strand.	1.154	1.039–1.281	0.007
CXCL1	ENSG00000163739	Chromosome 4: 73869393–73871308 forward strand.	1.121	1.019–1.233	0.018
CCL2	ENSG00000108691	Chromosome 17: 34255274–34257208 forward strand.	1.123	1.022–1.234	0.016
CXCL6	ENSG00000124875	Chromosome 4: 73836640–73849064 forward strand.	1.061	1.016–1.107	0.007
DRAM1	ENSG00000136048	Chromosome 12: 101877580–102012130 forward strand	1.234	1.011–1.506	0.039
LITAF	ENSG00000189067	Chromosome 16: 11547722–11636381 reverse strand.	1.676	1.208–2.325	0.002

Then, we constructed a TEX‐derived gene signature risk scoring model for survival prediction according to the expression of 12 TEX genes and using the multivariate Cox regression coefficient as the weight (termed 12TexSig), as follows: 12TexSig = expression value of CD79B*0.6062 + expression value of CKAP4*0.4571 + expression value of DUSP5*0.5388 + expression value of MTHFD2*(−0.4966) expression value of OGFR*0.3333 + expression value of SPON2*(−0.6398) + expression value of BANK1*0.2769 + expression value of CXCL1*(−0.2418) + expression value of CCL2*0.3836 + expression value of CXCL6*0.3741 + expression value of DRAM1*(−0.8747) + expression value of LITAF*0.5239. To accelerate the clinical transformation, we also constructed a nomogram to predict 1‐, 3‐ and 5‐year survival rates using these 12 genes (Figure 4B). With the 12TexSig, the risk score was computed for each patient in the TCGA cohort. All patients of the TCGA cohort were divided into a high‐risk group (*n* = 102) and a low‐risk group (*n* = 65) according to the optimal risk cut‐off value. The distribution of 12TexSig risk scores, gene expression profiles and survival status are shown in Figure 4C. Survival analysis indicated that patients in the high‐risk group had significantly shorter overall survival than those in the low‐risk group (HR = 0.282, 95% CI 0.186–0.424; log‐rank *p* < 0.001) (Figure 4D).

We next assessed whether the prognostic value of the12TexSig was independent of the clinical variable through multivariate Cox regression analysis. Results from the multivariate analysis indicated that the the12TexSig was significantly associated with overall survival when adjusted for age and gender (HR = 2.642, 95% CI 2.069–3.373, *p* < 0.001) (Figure 4E).

### 3.5 Independent validation of the 12TexSig for survival prediction

**FIGURE 5:**
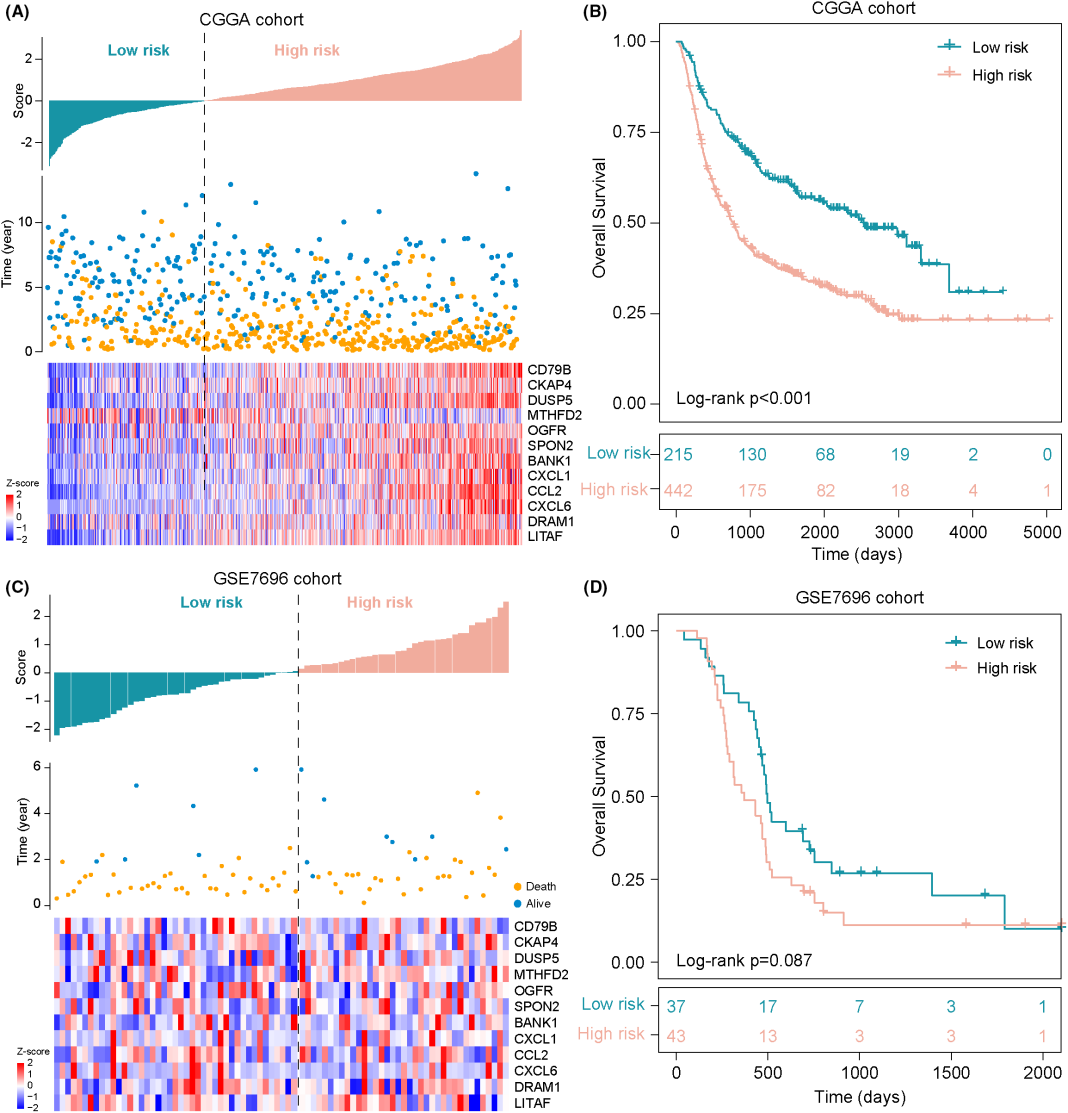
Independent validation of the 12TexSig for survival prediction in external cohorts. The distribution of 12TexSig risk scores, gene expression profiles and survival status in the TCGA cohort (A) and GSE7696 cohort (C). Kaplan–Meier survival curves of overall survival between two risk groups predicted by 12TexSig in the TCGA cohort (B) and GSE7696 cohort (D).

To test the robustness of the 12TexSig, we also examined the prognostic value of the 12TexSig in two external cohorts. We first tested the prognostic value of the 12TexSig in the CGGA cohort, in which the 12TexSig classified 693 samples into the high‐risk and low‐risk groups with significantly different survival. The distribution of 12TexSig risk scores, gene expression profiles and survival status are shown in Figure 5A. The overall survival of patients in the high‐risk group was significantly lower than in the low‐risk group (log‐rank *p* < 0.001) (Figure 5B).

When applying the 12TexSig to the GSE7696 cohort, we calculated the risk score for each patient of the GSE7696 cohort and categorized 80 samples into a high‐risk group (*n* = 43) and a low‐risk group (*n* = 37). The distribution of 12TexSig risk scores, gene expression profiles and survival status are shown in Figure 5C. Consistent with the findings described in the TCGA cohort, patients in the high‐risk and low‐risk groups showed significantly different outcomes. The survival time of the high‐risk group patients was shorter than that of low‐risk group patients (log‐rank *p* = 0.087) (Figure 5D).

## 4 DISCUSSION

In this study, we conducted an integrative multi‐omics analysis of T cell exhaustion in the tumour microenvironment of GBM patients, and identified two GBM subtypes characterized by distinct immunological, molecular and clinical characteristics. We subsequently evaluated the correlation between transcriptional profiles of TEX‐related genes and survival outcomes, and proposed and validated a TEX‐derived 12‐gene signature allowing for molecular classification and prognostic risk stratification.

There was increasing evidence that the tumour microenvironment, comprising cancer cells, stromal cells, blood vessels, nerve fibres, extracellular matrix and associated acellular components, play critical and various roles in carcinogenesis, cancer progression, and therapeutic outcome.^34^, ^35^ Immune cells have been known as significant components of the tumour microenvironment. Tumour‐infiltrating T cells are one of several essential components in the tumour immune microenvironment, and their states and abundances varied substantially in the tumour microenvironment across different cancer types reported in a pan‐cancer study.^36^ Although tumour‐infiltrating T cells are critical effectors of antitumor immune responses, they often become gradually exhausted with a dynamic process and developmental hierarchy.^19^ Recent advances in the tumour immune microenvironment of GBM have indicated that immunosuppressive cells dominate a wide variety of immune cell types leading to the highly immunosuppressive status in the glioblastoma tumour microenvironment.^37^ T cells in the tumour immune microenvironment play critical roles in the development and prognosis of GBM. For example, a study by Keskin et al. has found that prolonged survival of GBM patients is associated with increased T‐cell infiltration.^38^ However, T‐cell dysfunction and exhaustion have been well‐recognized as a hallmark of GBM.^22^ Therefore, in this study, we first characterized the expression pattern of TEX genes and classified GBM patients into different subgroups based on the expression of TEX genes. These two subgroups had heterogeneous TEX patterns and different survival outcomes. Notably, the prognosis of patients in the TEX‐C1 subgroup with an activated T cell activity status is worse than those in the TEX‐C2 subgroup with severe TEX status, suggesting a great need for clinical evaluation for TEX status to improve outcomes.

To meet this need, we used an integrated computational strategy to identify 12 key TEX genes with independent predictive power. Then, we integrated 12 essential TEX genes into a gene signature for scoring the prognosis risk of GBM patients. When testing the 12TexSig in different cohorts, the 12TexSig revealed consistently good performance in stratifying patients into significantly different prognosis groups, indicating the robustness and reliability of the 12TexSig in predicting outcomes. Furthermore, the results of the multivariate analysis also showed that the prognostic value of the 12TexSig is independent of clinical features. Of the 12TexSig, recurrent mutations of CD79B have been reported as the hallmark of primary central nervous system lymphomas.^39^ CKAP4 is an essential regulator of carcinogenesis, and its upregulation promotes the malignant progression of human gliomas and is associated with poor patient survival.^40^ The potential tumour‐suppressor role of MTHFD2 has recently been reported in GBM cells via the inhibition of ERK1/2 phosphorylation.^41^ Dysregulated chemokines in TME have been reported to favour the growth of tumours, including GBM.^42^ A recent study from Huang et al. reported that LITAF might enhance the radiosensitivity of glioma cells via the FoxO1 pathway.^43^

Several limitations should be noted in this study. First, this gene panel is tested only in limited patient cohorts and should be validated in more patient cohorts later. Second, the biological and molecular mechanism undying the TEX subtype should be explored in biological experiments.

In conclusion, our study has identified a novel 12‐gene signature derived from TEX‐related genes, providing accurate prognostic information for GBM patients. With further prospective validation, this signature could be a valuable biomarker to guide clinical decision‐making for GBM patients.

## AUTHOR CONTRIBUTIONS

**Yue‐hui Liu:** Data curation (lead); formal analysis (lead); methodology (lead). **Hong‐quan Jin:** Data curation (equal); formal analysis (equal). **Hai‐ping Liu:** Conceptualization (lead); funding acquisition (lead); writing – original draft (lead); writing – review and editing (lead).

## FUNDING INFORMATION

This study was supported by the fund for the 2022 Medical and Health Science and Technology Project of Health Commission of Inner Mongolia Autonomous Region of China (No. 202201461) and the Natural Science Foundation of Inner Mongolia Autonomous Region of China (No. LHNS08034).

## CONFLICT OF INTEREST STATEMENT

The authors declare that they have no interest.

## Supporting information


Table S1

Table S2

## Data Availability

Expression profiles and clinical information of three GBM cohorts used in this study were available from The Cancer Genome Atlas project (https://xena.ucsc.edu/), Chinese Glioma Genome Atlas (CGGA) database (http://www.cgga.org.cn/) and Gene Expression Omnibus under accession number GSE7696 (www.ncbi.nlm.nih.gov/geo/query/acc.cgi?acc=GSE7696).
